# Understanding the challenges to facilitating active learning in the resident conferences: a qualitative study of internal medicine faculty and resident perspectives

**DOI:** 10.3402/meo.v20.27289

**Published:** 2015-07-07

**Authors:** Adam P. Sawatsky, Susan L. Zickmund, Kathryn Berlacher, Dan Lesky, Rosanne Granieri

**Affiliations:** 1 Division of General Internal Medicine, Mayo Clinic, Scottsdale, AZ, USA; 2 Center for Health Equity Research and Promotion, VA Pittsburgh Healthcare System, Pittsburgh, PA, USA; 3 Division of Cardiology, School of Medicine, University of Pittsburgh, Pittsburgh, PA, USA; 4 School of Medicine, University of Pittsburgh, Pittsburgh, PA, USA; 5 Division of General Internal Medicine, School of Medicine, University of Pittsburgh, Pittsburgh, PA, USA

**Keywords:** medical education, graduate medical education, conference, lecture, active learning, lifelong learning

## Abstract

**Background:**

In the Next Accreditation System, the Accreditation Council for Graduate Medical Education outlines milestones for medical knowledge and requires regular didactic sessions in residency training. There are many challenges to facilitating active learning in resident conferences, and we need to better understand resident learning preferences and faculty perspectives on facilitating active learning. The goal of this study was to identify challenges to facilitating active learning in resident conferences, both through identifying specific implementation barriers and identifying differences in perspective between faculty and residents on effective teaching and learning strategies.

**Methods:**

The investigators invited core residency faculty to participate in focus groups. The investigators used a semistructured guide to facilitate discussion about learning preferences and teaching perspectives in the conference setting and used an ‘editing approach’ within a grounded theory framework to qualitative analysis to code the transcripts and analyze the results. Data were compared to previously collected data from seven resident focus groups.

**Results:**

Three focus groups with 20 core faculty were conducted. We identified three domains pertaining to facilitating active learning in resident conferences: barriers to facilitating active learning formats, similarities and differences in faculty and resident learning preferences, and divergence between faculty and resident opinions about effective teaching strategies. Faculty identified several setting, faculty, and resident barriers to facilitating active learning in resident conferences. When compared to residents, faculty expressed similar learning preferences; the main differences were in motivations for conference attendance and type of content. Resident preferences and faculty perspectives differed on the amount of information appropriate for lecture and the role of active participation in resident conferences.

**Conclusion:**

This study highlights several challenges to facilitating active learning in resident conferences and provides insights for residency faculty who seek to transform the conference learning environment within their residency program.

Under the Next Accreditation System, the Accreditation Council for Graduate Medical Education (ACGME) has established milestones to evaluate achievement of competency for medical knowledge (1). Within its common program requirements, the ACGME requires regular didactic sessions to achieve these milestones (2). Despite limited evidence that didactic conference attendance affects scores on national standardized exams (3–8), the main format for teaching residents remains the noon conference lecture series (9).

Medical educators have proposed the implementation of active learning formats to improve learning outcomes in residency training (10). Studies demonstrate increased medical student satisfaction and equivalent knowledge acquisition with methods such as case-based teaching and problem-based learning (11–13). The challenge arises when trying to apply these strategies within the residency environment. A few studies have shown benefit with the use of strategies to facilitate active learning in residency, including small groups, team-based learning, and problem-based learning (14–17). Yet, efforts to facilitate active learning in didactic sessions in residency are time consuming and require a lot of faculty time and effort (17). There are less time and resource-intensive strategies that can be implemented (18). There is little empirical data for many of these strategies, although one meta-analysis demonstrated a modest benefit of using an audience response system (19). Medical educators need to understand more about faculty teaching perspectives and resident learning preferences to efficiently and effectively apply adult learning theory to the residency learning environment.

Internal medicine residents at our institution are required to attend a variety of conferences, including regular didactics delivered in the lecture format. We previously explored resident learning preferences with the conference format, identifying that residents desire active learning (20). The purpose of this study was to explore faculty learning preferences within the conference format and their perspectives on facilitating active learning in resident conferences. Our goal was to identify challenges to implementing teaching strategies to enhance active learning in the residency didactic curriculum, both through identifying specific implementation barriers and identifying differences in perspective between faculty and residents on effective teaching and learning strategies.

## Methods

### Study context

We previously conducted seven focus groups with internal medicine residents (20). For this study, we conducted additional focus groups of internal medicine core faculty at the University of Pittsburgh to evaluate the challenges to implementing teaching strategies with the conference format that encourage active learning (21). The study team members had expertise in medical education, development and moderation of focus groups, and qualitative analysis. The study was approved by the Institutional Review Board of the University of Pittsburgh.

### Focus group participants

We invited faculty members to participate if they had a role in residency education, including associate program director, subspecialty education coordinator, or key clinical faculty.

### Focus group guide

We developed a focus group guide according to the described focus group methodology (22). The focus group guide contained questions on faculty learning preferences within the conference setting and perspectives of facilitating active learning during resident conferences. A draft of the guide was pilot-tested with general internal medicine fellows. Using feedback from the pilots, we refined and finalized the focus group guide.

### Focus group methods

We conducted three 1-h focus groups with core internal medicine residency faculty. A trained member of the University of Pittsburgh Qualitative Research Core (DL), who was independent from the residency training program, served as moderator. The principal investigator (AS) was present at each group as a note-taker. Notes were used for preliminary analysis and to guide subsequent focus groups. All focus groups were audio-recorded, and the moderator transcribed each focus group verbatim and de-identified the transcripts prior to analysis.

### Analysis

The transcripts were uploaded to ATLAS.ti version 6 (Scientific Software Development, Berlin, Germany), a computer software program to support the analysis of qualitative data. We used the ‘editing approach’ within a grounded theory framework to qualitative analysis developed by Crabtree and Miller (23, 24). The approach started with the AS and a qualitative methods expert (SZ) reviewing each transcript and developing a codebook through a process of open coding. Two trained coders then applied the codebook to the pilot transcripts to refine the codebook; they then independently applied codes to the focus group transcripts and adjudicated differences through discussion. Prior to the adjudication process, we used the individual coding files to calculate inter-coder reliability using the Cohen's κ statistic. The total mean kappa value for the assignment of codes was 0.78, what Landis and Koch define as ‘substantial’ agreement (25).

The research team reviewed the codes and quotations, and identified themes through an iterative process through the review of codes, quotations, and analytic memos as a research team. These themes were then organized into categories that helped to answer the research goals of identifying faculty learning preferences in the conference format and perspectives on how to facilitate active learning during resident conferences. These data were then compared to data previously obtained from the resident focus groups to identify similarities and differences in learning preferences that might inform the research goal. The research team outlined these categories and themes and chose exemplary quotations. We reviewed each theme and stopped data collection when thematic saturation was achieved.

## Results

Twenty core faculty participated in three focus groups; 12 (60%) were general internists and 8 (40%) were subspecialists. From our previous work, 41 internal medicine, medicine-pediatrics, preliminary, and transitional year residents participated in seven focus groups; 17 (41%) were PGY-1 and 24 (59%) were PGY-2 through PGY-4 residents. We identified themes within three domains: barriers to facilitating active learning formats, similarities and differences in faculty and resident learning preferences, and divergence between faculty and resident opinions about effective teaching strategies. There were no major differences identified among the three faculty focus groups.

### Barriers to facilitating active learning

Faculty members discussed the barriers to facilitating active learning during resident conference, including setting, faculty, and resident barriers (Table 1). Faculty members discussed the auditorium layout and resident behaviors (sitting in the back of the room, being interrupted by pagers) as physical barriers to facilitating active learning. They also discussed the amount of work it would take to reformat old lectures to include time for engagement and to formulate stimulating questions for discussion as barriers. Part of this stemmed from lack of knowledge and experience in facilitating active learning strategies, but there was also an underlying discomfort with giving up some control of the audience. Finally, there were concerns over the current residency environment, as active engagement was not part of regular conference structure or culture, and therefore change would require resident buy-in.

**Table 1:** Barriers to facilitating active learning strategies in resident conferences

Barriers	Faculty quotation
Setting barriers	
Large auditorium	‘The problem with trying to break it up and promote discussion is that there are about 50 people in the room, and you might want to encourage more interaction, but the more you ask, I just think the more difficult it will be for the residents…It just gives them more opportunity to feel anxious about public speaking’.
Seats organized in rows	‘The room is already set up in a way that is anti-interaction, like most lecture rooms are. All the chairs are in a straight row and everyone is facing forward, and most of the people sit in the back’.
Interruptions	‘If you want to develop groups that interact together, and people come in late and they leave early, they get paged, I think that's a challenge’.
Faculty barriers	
Formulating good questions	‘I think the hardest thing for people to do [in facilitating active learning] is to develop good questions’.
Reworking established lectures	‘There are a lot of people who do noon conference every year and they do the same topic, and then you are going to ask them to change it and it is going to be uncomfortable and it will take extra work’.
Giving up control of the learning environment	‘You lose control. The more interactive, the more you let the audience go, and if you have teaching goals you want to accomplish, that could work out but it is just a little bit more risky’.
Lack of training	‘You will need faculty development or guidelines. It would be a fair amount of training for someone who hasn't done it before’.
Resident Barriers	
Residency culture and resident buy-in	‘… changing the residency culture and getting the residents to buy in, participate and stay—you wouldn't want them to know they now have to participate and have them not come’.

In this table, we outline the barriers that faculty describe for facilitating active learning strategies in resident conferences.

### Similarities and differences in learning preferences

**Fig. 1:**
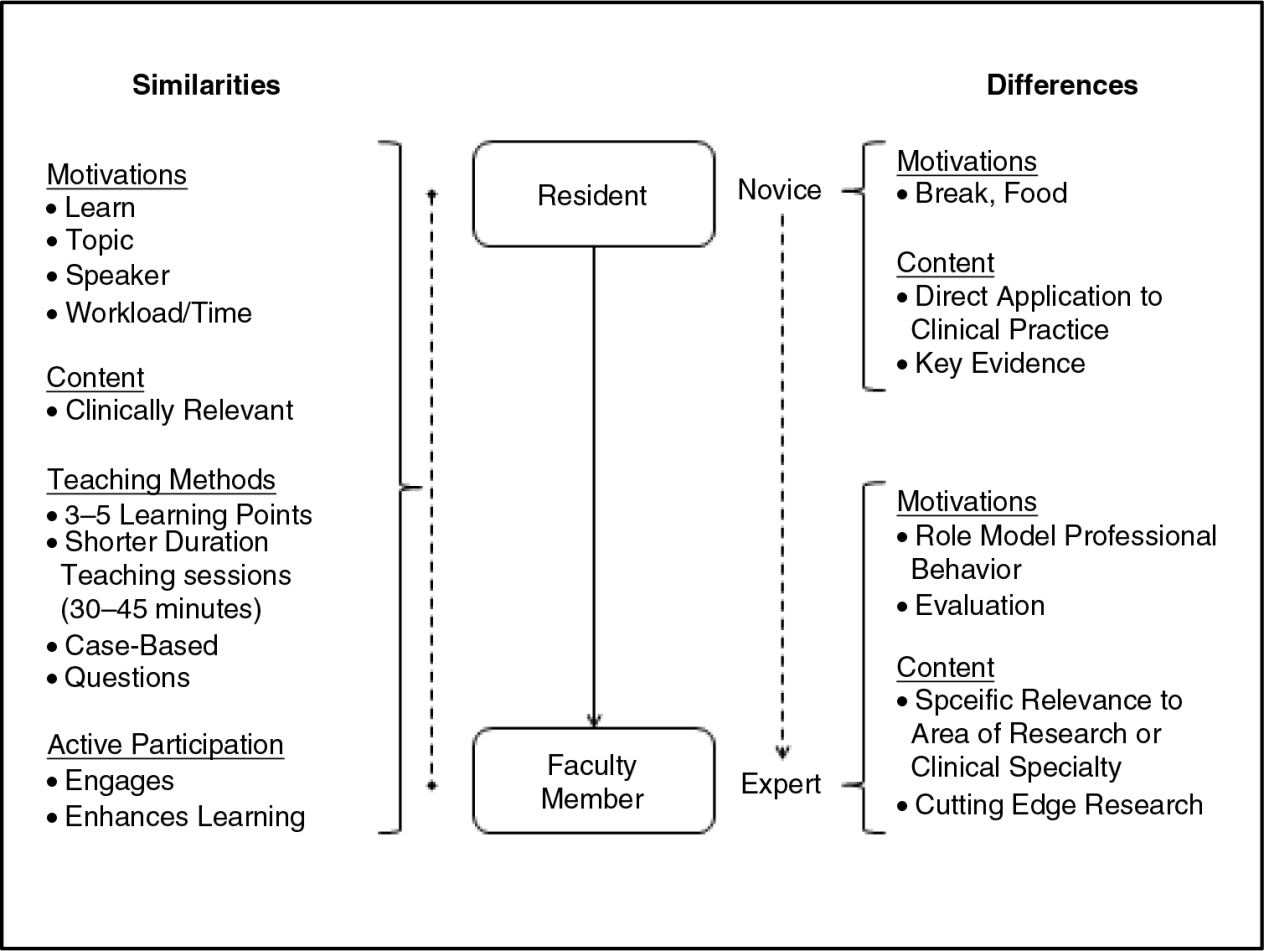
Similarities and differences in learning preferences between residents and faculty. The left side of the figure outlines the similarities between residents and faculty members in motivations for attending or not attending conferences, desired content, preferred teaching methods, and perspectives on active participation. The right side of the figure outlines the main differences between residents and faculty members in motivations for attending or not attending conferences and desired content.

We compared themes between residents and faculty to examine similarities and differences in learning preferences within the conference format. While there were many similarities, the differences between residents and faculty help to highlight areas for improvement when teaching residents in the conference format (Fig. 1).

The main differences between faculty and residents included motivations for attending conferences and the type of content that was desired. For the theme of motivation, residents discussed attending conferences to get a break from their clinical work and obtain food. Faculty discussed attending conferences with residents to role model professional behaviors. They also discussed their role in curriculum evaluation for the conferences that they were responsible for coordinating, but did not personally present. Residents did not discuss attending conferences to assess the quality of the conference.

Another difference was the desired content for conferences. Residents were interested in understanding the evidence that guides current clinical practice, including landmark articles. They desire content that has direct application to their clinical practice and is at the level of a resident or general internist. Faculty members discussed attending conferences on topics of specific relevance to their area of expertise or research (often a specialty or subspecialty interest), having a more specific focus than residents. Faculty also discussed a desire to hear about ‘cutting-edge’ research that may not be immediately applicable to their current clinical practice, but may affect future practice. One faculty member summarized this difference in the level of clinical content: ‘If someone is going to give a talk on “which should you use: warfarin or dabigatran for atrial fibrillation,” I might go to that because I haven't figured that out yet, whereas a resident may still need to learn how you manage atrial fibrillation’.

A comparison of the comments regarding Medical Grand Rounds also highlighted these differences well. Residents found the topics esoteric and not applicable to their clinical practice, while a faculty member expressed the opposite view: ‘Grand Rounds should be the state of the art knowledge, not necessarily something you can walk out with and apply to the patient you are seeing tomorrow’.

### Divergent perspectives of effective teaching strategies

There were differences between faculty member's approaches to teaching and resident's learning preferences that highlighted challenges with using active teaching strategies in the conference format. The two main tensions centered on the appropriate amount of information to be presented in a conference and the appropriate use of active engagement during a conference.

#### Amount of information

Although both faculty members and residents discussed that lecturers should present a limited number of learning points, faculty discussed the pressure to provide comprehensive information. One faculty member stated: ‘You don't give a lot of talks, so you feel pressed to provide all the key information even if you know it's not the most effective way, because you don't want them to never have heard it’. Faculty members’ perceived need to provide comprehensive information in resident conferences directly opposed the stated learning preferences of both groups.

#### Engagement

Both groups acknowledged that active participation improves investment in the learning process and enhances overall learning, but faculty discussed the difficulty they encounter when trying to motivate residents to interact in conferences. One faculty member highlighted: ‘You can make the lecture interactive, but then what happens a lot of times you don't get any response from the residents’. The difficulty that faculty perceive seems to contradict the stated learning preferences of residents.

While residents appreciated active engagement during conferences, they discussed at length the importance of creating a safe environment to encourage interaction and participation. Residents did not like being ‘called on’ or ‘pimped’ in the conference setting: ‘I think that makes some people feel uncomfortable and I don't think that picking on someone is the way to go about it. I think there are different ways to make it interactive’. Residents discussed effective strategies to create a safe environment for participation, including setting expectations ahead of time, asking questions in a nonthreatening way, not putting residents on the spot to answer questions, and creating a culture where large group discussion is expected.

One method discussed by both groups to increase audience participation was the use of an audience response system. Faculty members felt that it was beneficial in engaging residents in the lecture. One faculty member stated: ‘I think that's the strength of the audience response system that it makes it safe to respond’. While residents agreed that audience response system makes it safer to answer questions in a larger audience, they questioned its effectiveness at engaging them in learning. One resident argued: ‘I guess it's better than having no participation. I mean, it's some interaction. I don't know how dynamic it is’.

### Area for exploration

While critically evaluating resident conferences, interesting questions arose among the faculty. Most prominent was:

Should we have noon lectures at all? From what everybody is saying, one conclusion that you could draw is that giving lectures in this setting is a bad idea. Maybe the answer is that we shouldn't have lectures.

## Discussion

We explored challenges for faculty to facilitating active learning in resident education conferences. First, faculty identified very specific setting, faculty, and resident barriers to active learning that need to be overcome. Second, while we found many similarities in faculty and resident learning preferences, we identified differences in motivations for conference attendance and desired content. These differences need to be understood by faculty, when planning conferences and seeking to facilitate active learning. Third, when comparing faculty perspectives on teaching to resident learning preferences, there were two main divergent themes: 1) faculty members feel pressure to provide comprehensive information in conferences while residents feel overloaded with information, and 2) faculty members have difficulty getting residents to actively participate in conference while residents state a desire active participation in a safe environment. These overt barriers and subtle differences all present challenges to facilitating active learning in resident conferences.

As residency program leadership seeks to balance ACGME requirements with the changing face of residency education, medical educators need to create efficient and effective learning opportunities. Our findings provide practical guidance to aid in this change. First, faculty need to understand the unique learning preferences of residents and craft learning opportunities that provide clinically relevant level-appropriate information and evidence. They cannot expect that lectures prepared for other levels of learners will meet the needs of residents.

Second, faculty need to overcome their internal pressure to provide complete and thorough information in the lecture format. This study illustrated that faculty feel that if they do not provide information, the residents will not learn that information. We know that residents are exposed to a variety of learning environments and often learn best from clinical encounters and self-directed learning episodes (8, 26,27). The primary goal of resident conferences should change from information dissemination to information application and clinical reasoning.

Third, while residents desire active learning, faculty described difficulty with facilitating active learning, even amongst seasoned educators. Their main concern was giving up control of the learning environment. Malcolm Knowles invites teachers of adults to move from being content transmitters to becoming facilitators of learning; he describes the uncomfortable nature of this process, as one ‘divest[s] [themselves] of the protective shield of authority and expose [themselves]’ (28). Faculty members should continue to pursue active engagement in conferences and explore means for creating a safe environment to facilitate resident participation.

More research needs to be done to answer the final question: ‘Should we have lectures at all?’ Until then, residency programs need to optimize the learning opportunities for their residents. Barriers to facilitating active learning can be accomplished by supporting faculty through providing training, structure, and feedback on teaching. There are several structured formats for facilitating active learning, such as problem-based learning and team-based learning, which have been adapted to residency education (15, 16). While these have shown promise, they require a large amount of programmatic change (17). Also, because they have been developed in other situations, they may not fully suit the residency training environment. We used the results of this study to build an ACTIVE teaching format that gave faculty a structure for facilitating active learning while needing minimal faculty development time and effort (29). This ACTIVE format was centered on 3–5 focused learning points, clinical questions, small group break-out discussions, and frequent summarization (29). This is only one example, and further work needs to be done to identify easily-implemented strategies to support residency faculty in facilitating active learning.

Several factors may limit the transferability of our findings to other residency programs. First, the primary author played a large role in data collection and analysis. To address this, we used an independent moderator and a secondary coder and achieved strong inter-coder reliability. Second, our study was conducted at a single institution and included only internal medicine faculty and residents and may not be applicable to other disciplines. Third, our internal medicine residency is part of the ACGME's Education Innovation Project, a factor that may affect the views of our residents toward continued innovation. Although our program is large, university-based and may not reflect the spectrum of environments in other residency training programs, it is similar to all that must meet ACGME requirements for formal didactic sessions.

## Conclusions

As medical educators seek to improve resident education within the conference format by facilitating active learning, it is essential to understand the challenges to implementing these strategies. We outlined some barriers to implementing active learning formats. We also identified differences between residents and faculty learning preferences, as well as perspectives on resident learning, which can provide insights to providing opportunities for learning in resident conferences. In understanding these challenges, residency faculty can transform the conference learning environment to truly engage learners.
